# NIRS and Aquaphotomics Trace Robusta-to-Arabica Ratio in Liquid Coffee Blends

**DOI:** 10.3390/molecules27020388

**Published:** 2022-01-08

**Authors:** Balkis Aouadi, Flora Vitalis, Zsanett Bodor, John-Lewis Zinia Zaukuu, Istvan Kertesz, Zoltan Kovacs

**Affiliations:** 1 Department of Measurements and Process Control, Institute of Food Science and Technology, Hungarian University of Agriculture and Life Sciences, 14-16. Somlói Street, H-1118 Budapest, Hungary; aouadi.balkis@phd.uni-mate.hu (B.A.); vitalis.flora@phd.uni-mate.hu (F.V.); zsanett.bodor93@gmail.com (Z.B.); kertesz.istvan@uni-mate.hu (I.K.); 2 Department of Dietetics and Nutrition Faculty of Health Sciences, Semmelweis University, 17. Vas Street, H-1088 Budapest, Hungary; 3 Department of Food Science and Technology, Kwame Nkrumah University of Science and Technology (KNUST), Kumasi 00233, Ghana; zaukuu.jz@knust.edu.gh

**Keywords:** coffee, NIRS, aquagrams, chemometrics, authentication, PCA, PCA-LDA, PLSR

## Abstract

Coffee is both a vastly consumed beverage and a chemically complex matrix. For a long time, an arduous chemical analysis was necessary to resolve coffee authentication issues. Despite their demonstrated efficacy, such techniques tend to rely on reference methods or resort to elaborate extraction steps. Near infrared spectroscopy (NIRS) and the aquaphotomics approach, on the other hand, reportedly offer a rapid, reliable, and holistic compositional overview of varying analytes but with little focus on low concentration mixtures of Robusta-to-Arabica coffee. Our study aimed for a comparative assessment of ground coffee adulteration using NIRS and liquid coffee adulteration using the aquaphotomics approach. The aim was to demonstrate the potential of monitoring ground and liquid coffee quality as they are commercially the most available coffee forms. Chemometrics spectra analysis proved capable of distinguishing between the studied samples and efficiently estimating the added Robusta concentrations. An accuracy of 100% was obtained for the varietal discrimination of pure Arabica and Robusta, both in ground and liquid form. Robusta-to-Arabica ratio was predicted with R^2^CV values of 0.99 and 0.9 in ground and liquid form respectively. Aquagrams results accentuated the peculiarities of the two coffee varieties and their respective blends by designating different water conformations depending on the coffee variety and assigning a particular water absorption spectral pattern (WASP) depending on the blending ratio. Marked spectral features attributed to high hydrogen bonded water characterized Arabica-rich coffee, while those with the higher Robusta content showed an abundance of free water structures. Collectively, the obtained results ascertain the adequacy of NIRS and aquaphotomics as promising alternative tools for the authentication of liquid coffee that can correlate the water-related fingerprint to the Robusta-to-Arabica ratio.

## 1. Introduction

The worldwide appeal of coffee, consumed not only as a functional beverage, but also as a provider of unique cultural experiences, stems from its distinct organoleptic features. These criteria are mostly defined by the respective geographic and varietal origin as well as the brewing processes.

While genus *Coffea* exists under numerous varieties, *Coffea arabica* and *Coffea canephora* are the two commonly consumed ones [1]. Arabica, the priciest of the two and marketed as having the higher quality grade, has been a prime target for fraud, propelled by the potential economic gains [2].

On the consumer front, rising demands for safe products that impart the desired nutritional and sensory values and authentically state the actual ingredients present are driving a palpable sense of responsibility shared by the food industry operators and academia alike. With regulations stipulating no more than 1% of foreign materials in coffee [3], this challenge becomes even more daunting.

Upon adulteration, affecting the flavor profile of coffee is not the sole repercussion on coffee quality. Other reported aspects comprise alterations of the antioxidant capacity and reduction of the levels of bioactive compounds [4].

Although a common practice, mixing the two coffee varieties, unless otherwise stated, can be considered as a milder type of coffee forgery. Innumerable studies investigated the efficiency of detecting such occurrences. Wermelinger et al. [5], for instance, attempted the quantification of the Robusta fraction in a coffee blend via Raman spectroscopy. Mixtures with Robusta contents of 5, 10, 25, 33, 50, and 75% *w*/*w* were classified. Kahweol, exclusively present in Arabica beans, enabled the discrimination of the two extracted lipid fractions of Arabica and Robusta with a detection limit ranging from 4.9 to 7.5% *w*/*w*. The higher the content of Robusta, naturally richer in unsaturated fatty acids, the greater the shift of the peak once situated at 1665 cm^−1^ (6006 nm).

For Schievano et al. [6], nuclear magnetic resonance (NMR) was the method of choice for authenticating coffee blends by quantifying 16-O-methylcafestol (16-OMC). The study accurately detected Robusta, at concentrations below 0.9%, with detection and quantitation limits of 5 and 20 mg per kg, respectively. Pure Arabica was equally 100% distinguished from Robusta-Arabica mixtures. Other suggested discriminators consisted of fatty acids, tocopherols, or sterols. These, however, necessitate additional extraction and separation operations as suggested by Schievano et al. [6].

Similarly, Milani et al. [3] used NMR to authenticate coffee adulterated with barley, corn, coffee husks, soybean, rice, and wheat added in 50% *w*/*w* proportion to pure coffee. Soft independent modelling by class analogy (SIMCA) provided 100% correct classification for both training and prediction sets and limits of detection of 0.31–0.86% in medium as well as in dark roasted coffee were obtained.

The differential electronic nose was employed by Brudzewski et al. [7] in analyzing Arabica coffee adulterated with 10, 20, 30, 40, 50, 60, 70, 80, and 90% Robusta. With an average error of 0.21%, the adopted 30-fold cross-validation model permitted the recognition of all mixtures.

For Pizarro et al., the approach differed, where calibration models developed from near-infrared spectra produced prediction models with root mean square error of prediction (RMSEP) of 0.79%. The added Robusta ranged from 0 to 60% *w*/*w* [8]. The methodology followed by Spaniolas et al. [9], consisted in differentiating the two varieties based on a lab-on-a-chip system and a limit of detection of 5% was achievable.

Other forms of adulteration involve the addition of both corn and soybean. Such cases were studied by Arrieta et al. and Daniel et al. [10,11], who deployed the voltametric electronic tongue and capillary electrophoresis-tandem mass spectrometry, respectively. Although efficient (R^2^ of 0.973 and 0.941 for the prediction of corn and soybean in case of e-tongue), these techniques demand high levels of technicity and relatively lengthy processing.

Combined with multivariate curve resolution (MCR), near infrared hyperspectral imaging was utilized to determine coffee husks, soil, wood sticks, and roasted corn kernel powders added in quantities of 1–40% [12]. Quantitative models with absolute errors not exceeding 4% were obtained.

Roasted barley, rice, and wheat powders are also some of the low-cost additions made to pure Arabica for increased profit margins. Song et al. [13] applied high performance liquid chromatography (HPLC) to quantitatively analyze coffee blends containing 1, 2, 3, 4, 5, 10, and 20% *w*/*w* of these powders. For the purpose of the study, monosaccharides, nicotinic acid, and trigonelline served as chemical indicators of the authenticity of coffee. Of all studied indices, glucose aided the most in the discrimination of pure and adulterated Arabica. The corresponding discrimination limit was 1% *w*/*w* with significant difference in ANOVA (*p* < 0.05).

Coffee quality analysis has mainly focused on beans and powders and only a few studies used aqueous coffee solutions. Amongst those who attempted to do so, Suhandy and Yulia intentionally added 10–60% of Robusta to Arabica and collected the UV-visible spectra of the aqueous solutions in the 200–400 nm range. They proved that the selection of specific intervals as a basis for building the partial least squares (PLS) models enhances the performance of the model, resulting in a ratio prediction to deviation (RPD) of 2.15 [14].

By conducting the present study, one of our objectives was to assess the applicability of aquaphotomics, as an innovative NIR-based approach in pinpointing potential blending of the two coffee varieties.

With demonstrated efficiency in a panoply of applications, aquaphotomics offers a holistic approach to the study of biosystems and the analysis of food matrices [15]. Aquaphotomics-related studies have substantiated that placing an emphasis on the water molecular system can in fact be an alternative to other more laborious techniques. In a sense, by applying various perturbations, structural changes of water species are induced and thus can be reflective, by comparison to control samples, of the state of the studied matrix. Most importantly, it brings about a new perspective regarding one of the commonly encountered limitations of conventional analysis: water.

This feature has been already used to track changes induced by cheese ripening [16], to screen water’s quality in the presence of certain contaminants [17], to elucidate yoghurt fermentation mechanisms [18], to authenticate honey [19], and many more applications [20,21,22,23]. With regards to coffee analysis, this approach could prove particularly beneficial in case of the shortage of beans, the absence of technically qualified coffee quality assessors as well as the insufficiency of the chemicals used for other sophisticated methods. The practical implications of the study could be particularly promising in cases where the authentication and detection of the adulteration is not possible in powder form namely with the expansion of the ready-to-drink (RTD) coffee market.

The aim of our research was to authenticate coffee both in its ground and liquid states using conventional NIRS and aquaphotomics. To do so, a comparative assessment of the quantification accuracy of Robusta-to-Arabica ratio in mixtures containing 0.5–35% of Robusta was conducted. Determining the impact such blending has on the respective water spectral pattern of the studied samples was also one of the prime objectives of our study. The obtained performances were evaluated by the inclusion of marketed blends of different varietal composition and geographical origin throughout the analysis steps.

## 2. Results and discussion

### 2.1. Varietal Discrimination of Pure Ground Coffee

A primary step consisted in assessing whether or not the applied method could discriminate between the pure varieties of ARA1, ARA2, ARA3, ROB1, ROB2, and ROB3 in the form of ground coffee.

**Figure 1:**
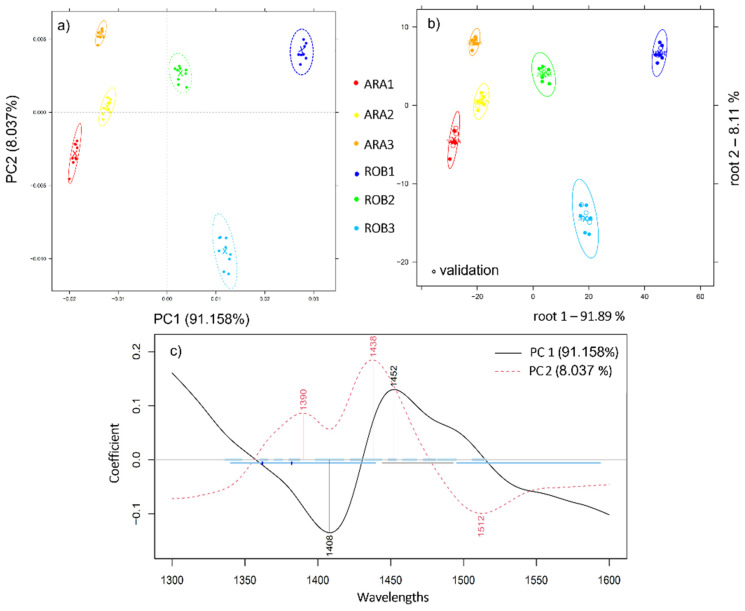
(**a**) Principal component analysis (PCA) on pure arabica (ARA1, ARA2, ARA3) and Robusta (ROB1, ROB2, ROB3) ground coffee samples in the wavelength range of 1300–1600 nm, N = 54, Savitzky-Golay smoothing (window size 19) and MSC; (**b**) PCA-LDA classification plot of pure Arabica (ARA1, ARA2, ARA3) and Robusta (ROB1, ROB2, ROB3) ground coffee samples, N = 54; (**c**) The respective PCA loading plot.

Analysis of pure ground coffee samples of differing varieties by means of principal component analysis, as showcased in Figure 1, demonstrates a pattern of separation along the axis of PC1, which together with PC2 accounts for 99% of the data variability. The efficacy of NIRS in terms of separating the samples was not only based on their respective varieties, but also on their provenance as the different samples came from different sources: Brazil (ARA1), Columbia (ARA2), Ethiopia (ARA3), Vietnam (ROB1), Uganda (ROB2), and India (ROB3). This trend suggests the compositional variability within each of the evaluated varieties. Indeed, studies have shown the role of geographical origin in conferring a specific chemical composition to coffee. This is in accordance with the findings reported by Giraudo et al. [24], who proved that intra-varietal differences of coffee beans originating from different countries and continents can be traced by their respective NIR spectral patterns. According to the corresponding loadings vector (Figure 1c), the wavebands 1390, 1408, 1438, 1452, and 1512 nm contributed the most to this separation. The PCA-LDA model scores yielded 100% recognition and prediction of the pure Arabica and Robusta varieties.

### 2.2. Near Infrared Analysis of Ground Coffee Mixtures

**Figure 2:**
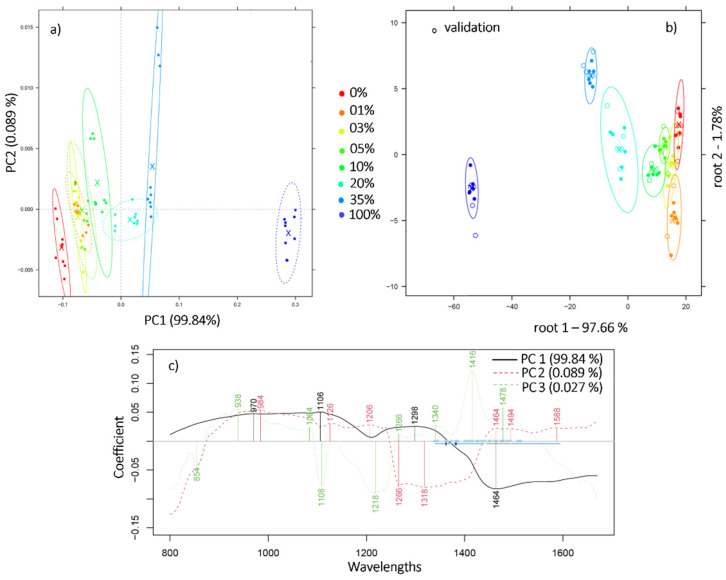
(**a**) PCA scores plot reveals separation along PC1 axis of the mixtures containing Robusta concentration in the range 1%–35%, N = 72, wavelength range 800–1670 nm, Savitzky-Golay smoothing (window size 19) and MSC; (**b**) PCA-LDA classification plot of the robusta adulterated ground coffee in the concentration range 1%–35%, wavelength range 1300-1600 nm, N = 72; (**c**) PCA loadings plot.

For the remainder of our study, we focused on the mixtures prepared by mixing the pair (ARA3, ROB3). To determine if a recognizable pattern is ascribable to the adulterated Arabica depending on the added Robusta, principal component analysis was performed in the 1st overtone (1300–1600 nm), 2nd overtone (800–1100 nm), as well as in the truncated spectral range of the instrument, 800–1670 nm. The model illustrating the most distinctive pattern was obtained in the range 800–1670 nm using the smoothed and MSC pretreated spectra (Figure 2a). According to the loadings plot (Figure 2c), the wavelengths responsible for the variance in the data are mostly those located at 970, 1106, 1126, 1266, 1298, 1318, and 1464 nm. Previous studies have attributed bond vibrations at 1126 nm of the 2 × C-H stretching and 2 × C-H deformation and (CH2)n C-H stretching second overtone to coffee fatty acids and chlorogenic acid (CGA) [25]. Indeed, these constituents have already proven to be good discriminators of the varietal origin of coffee [26].

Relying solely on the visual inspection of the separated samples, the truncated range 800–1670 nm served better for the pattern recognition of the mixtures with PCA. The analysis of the samples by means of linear discriminant analysis, however, proved better when performed at the first overtone 1300–1600 nm. An accurate recognition and prediction of 95.87% and 94.45% were obtained, respectively, using the raw spectra. The misclassifications occurred mainly between sample pairs (1% and 3%; 3% and 5%) whereas those comprising at least 10% Robusta were 100% accurately classified.

**Figure 3:**
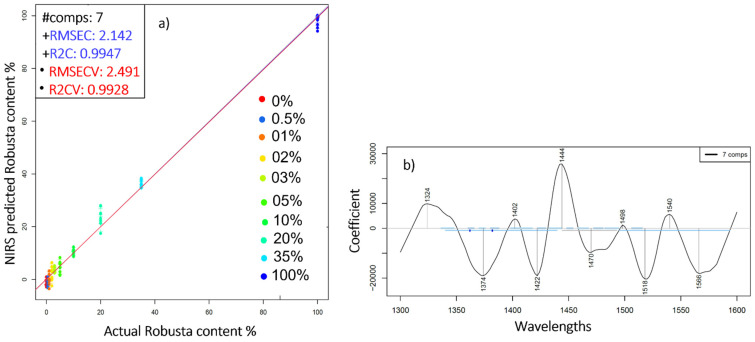
(**a**) PLSR analysis of the ground coffee mixtures derived from the smoothed (Savitzky-Golay smoothing (window size 19)) first derivative spectra for the prediction of added Robusta (% *w*/*w*); (**b**) The respective regression vector in the range 1300–1600 nm.

Once the mixtures were correctly classified, PLSR models were built in order to assess the feasibility of near infrared spectroscopy in predicting the Robusta to Arabica ratio. By leaving one group out (three consecutive scans of the same replicate) cross-validation, the model built on the smoothed first derivative of the spectra enabled a coefficient of determination (R^2^CV) of 0.99 and an error (RMSECV) of 2.4% (Figure 3a). Similar results (R^2^ > 0.99 and RMSE below 1.2% *w*/*w*) were found when evaluating Arabica-Robusta mixtures in the range of 0–60% [8].

The corresponding regression vector showcases the most significant wavelengths in terms of accurately determining the added Robusta. These peaks are located at 1324, 1374, 1402, 1422, 1444, 1470, 1498, 1518, 1540, and 1556 nm (Figure 3b). Prior studies have assigned wavelengths in the 1400–1600 nm range to some typical components of coffee, such as caffeine, sugar, and chlorogenic acids [27]. The addition of Robusta, naturally richer in chlorogenic acid and caffeine content [28], could explain the prominence of these particular wavebands when predicting the added Robusta.

**Figure 4:**
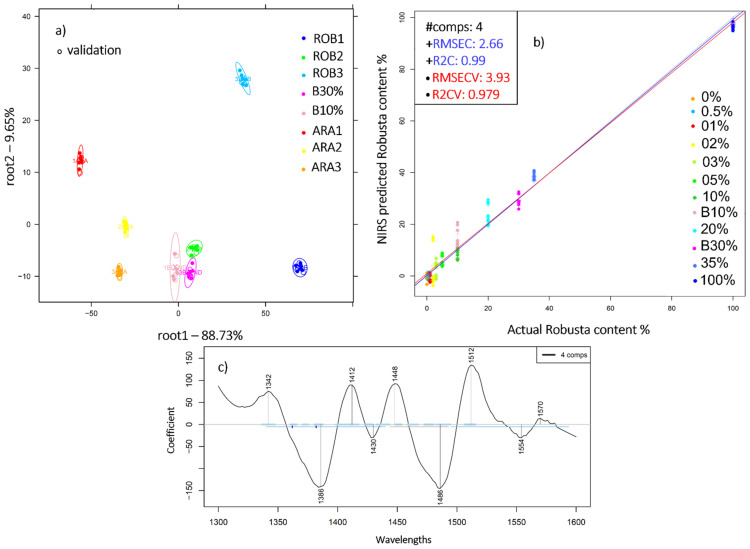
(**a**) PCA-LDA Classification of pure ground Arabica (ARA1, ARA2, ARA3), Robusta (ROB1, ROB2, ROB3) and marketed blends B10%, B30% in the range 1300–1600 nm, N = 72, spectral pretreatment: Savitzky-Golay smoothing (window size 19) and 1st derivative; (**b**) PLSR analysis of the ground coffee mixtures and B10% and B30%, spectral pretreatment: Savitzky-Golay smoothing (window size 17) and SNV; (**c**) The respective regression vector in the 1300–1600 nm.

The PCA-LDA classification and PLSR prediction of the Robusta-to-Arabica ratio were also performed on the marketed blends B10% and B30%. Figure 4a illustrates the obtained results where B10% and B30% were discriminated from the pure Arabica and Robusta ground coffee samples with 100% accuracies of recognition and prediction. The regression model built to predict the Robusta content and cross-validated by leaving three consecutive scans of each replicate at a time enabled an estimation of added Robusta with R^2^CV and RMSECV values of 0.97 and 3.93% *w*/*w*, respectively (Figure 4b). This slight difference compared to the model constructed only on the mixtures could be due to the different composition of the marketed blends B10% and B30%, obtained by combining other Arabica and Robusta varieties, from a geographical origin other than that of ARA3 and ROB3.

### 2.3. Near Infrared Analysis of Pure Liquid Coffee Extracts

**Figure 5:**
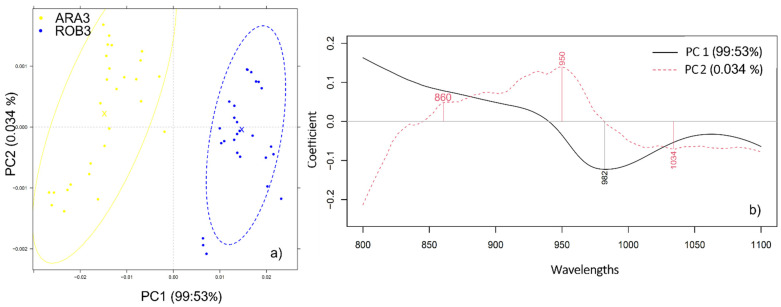
(**a**) PCA analysis applied to the spectra of pure Arabica (ARA3) and Robusta (ROB3) liquid extracts in the 800–1100 nm range, N = 54, spectral pretreatment: Savitzky-Golay smoothing (window size 19) and MSC; (**b**) The respective PCA loadings plot.

Performing principal component analysis on the pure Arabica (ARA3) and Robusta (ROB3) liquid extracts in the short wavelength range of 800–1100 nm revealed a pattern of separation into two respective clusters depending on the coffee variety (Figure 5). Combined, PC1 and PC2 accounted for more than 99% of the data variance and the bands contributing the most to this separation were positioned at 950, 982, and 1034 nm.

**Figure 6:**
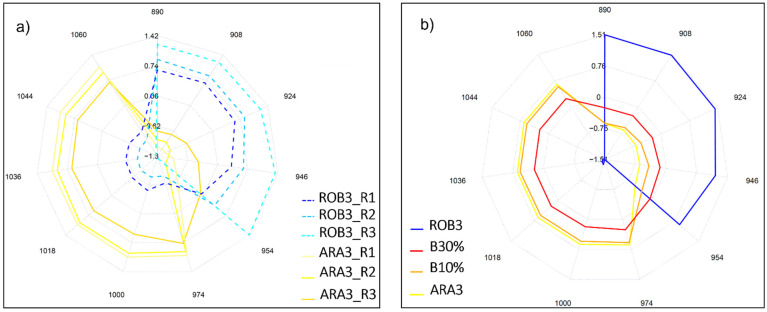
(**a**) Aquagram representation of the spectral pattern of pure Arabica and Robusta liquid extracts at the second overtone region (800–1100 nm), R1, R2, R3 denote replicates, N = 9 each; (**b**) Water spectral pattern of marketed blends B10% and B30%, pure Arabica (ARA3) and pure Robusta (ROB3), N = 27 each.

Next, absorbances of ARA3 and ROB3 aqueous samples as projected on the aquagram were investigated in 12 characteristic wavelength ranges in the 2nd water overtone in NIR region. What the aquagram accentuated is that Robusta coffee extracts, contrarily to Arabica, were majorly characterized by water molecules that are structured into water shells (908 nm), V1 and V2 bonded water while Arabica has high hydrogen bonded water structures (1060 nm) and is rich in water clusters with two, three, and four hydrogen bonds (1018, 1036, and 1044 nm) (Figure 6a). Wu et al. [29] are among those who investigated the compositional analysis of milk in the short NIR wavelength range (800–1050 nm) and reported the potential assignment of the 1018 and 1042 nm to the interaction of fat–water. The fact that Arabica is naturally richer in fat content can explain the high absorbance observed at these bands [30].

The incorporation of marketed blends B10% and B30% into the aquagram calculation is presented in (Figure 6b). Notably, the resulting water spectral pattern followed a logical sequence. Out of the two studied blends, the one with the highest Arabica content (B10%) had a similar pattern to pure Arabica, with slightly lower absorbance values. When the percentage of Robusta increased, as is the case of B30% blend, higher absorbances in the wavelength range of 890–954 nm were emphasized.

**Figure 7:**
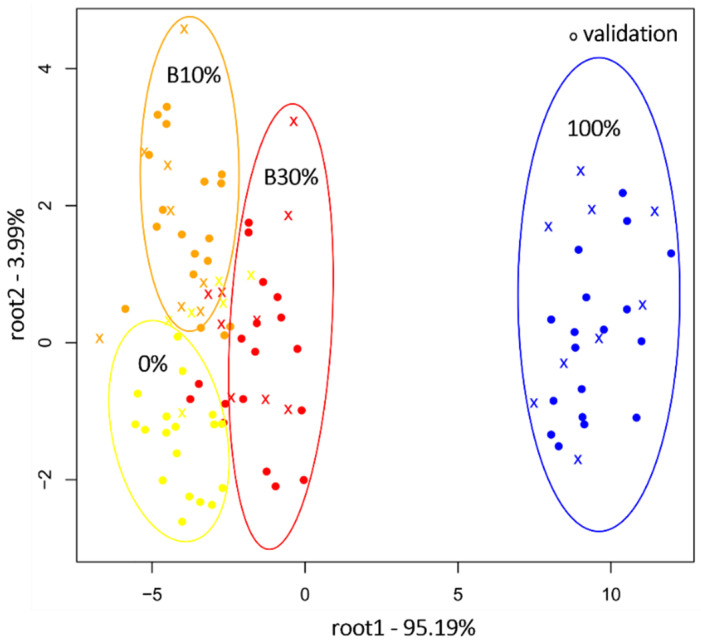
PCA-LDA classification of marketed blends B10% and B30%, pure Arabica (0%) and pure Robusta (100%) at the second overtone region (800–1100 nm), N = 108, NrPCs = 18.

Likewise, PCA-LDA was proven performant when assigning the samples ARA3, B10%, B30%, and ROB3 to their specific classes, with an accurate recognition of 91.26% of the samples while at the prediction level 83.39% were correctly categorized. The separation is most apparent along the axis of the first discriminant factor (Figure 7). The misclassifications occurred mostly between samples with proximate composition. Thus, B10% was primarily misclassified in 14.78% of the cases to the group 0% (ARA3) while B30% was identified as B10% in 11.11% of cases.

### 2.4. Near Infrared Analysis of Liquid Coffee Mixtures

When all adulteration levels were considered, LDA models destined at regrouping the samples into their corresponding groups enabled a 100% recognition of the different mixtures, and the prediction rate amounted to 71.32%. The following table recaps where misclassification occurred presumably due to the low Robusta concentrations or to the proximity of certain levels (Table 1). The classification was even less efficient when the model blends (B10%; B30%) were included into the construction of the predictive model (55.58% prediction rate). The blend B10% was misidentified as belonging to the group containing 5% Robusta in 11.14% of the studied cases and misclassified with 3.67% to the following groups: B30%, 35%, 20%, 10%. B30%, on the other hand, was wrongly categorized as belonging to the groups B10%, 35%, and 3% in 7.44% of the cases. While the blending ratio of these model blends fits into the range covered by our study, their heterogenous composition could have an effect on the classification accuracy. Indeed, the effect of the blend composition on the accuracy of the classification model has already been proven by Tavares et al. [31], who, basing their study on the analysis of the lipid extracts by HPLC, proved that proportions as high as 10% of maize and 20% of coffee by-products are required to identify the adulteration of coffee by means of PCA and LDA.

**Figure 8:**
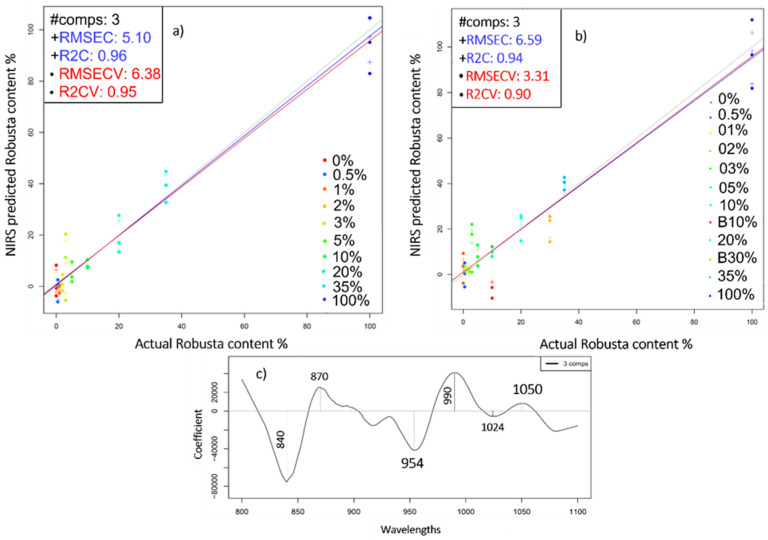
(**a**) Y-fit graph of the prediction of Robusta concentration in liquid mixtures N = 30; (**b**) PLSR analysis on the dataset containing marketed blends B10% and B30% and the mixtures, N = 36; (**c**) Regression vector of the predictive model (**b**) in the 800–1100 nm range. Spectral pre-processing: Averaging consecutive scans, Savitzky-Golay smoothing (window size 19), 1st derivative.

Averaging the consecutive scans and the parallel spectra of each of the studied mixtures and those of the controls (pure Arabica and pure Robusta) was proven effective when it comes to improving the accuracy of the predictive PLSR model. The optimal cross-validated model was the one built in the second overtone region, 800–1100 nm, and was characterized by R^2^CV and RMSECV values of 0.95 and 6.35% *w*/*w*, respectively (Figure 8a). Again, the blends lowered the accuracy of the regression model (R^2^CV = 0.9). The most prominent wavelengths corresponded to 840, 870, 954, and 990 nm (Figure 8c). Interestingly, the band situated at 954nm was already proven relevant when differentiating pure Arabica and Robusta based on their water spectral patterns (Figure 6). Similar results (R^2^ = 0.95) were obtained by Núñez et al. [32] when examining the HPLC-UV fingerprints of brewed Arabica coffee containing Robusta in proportions ranging from 15% to 85%.

**Figure 9:**
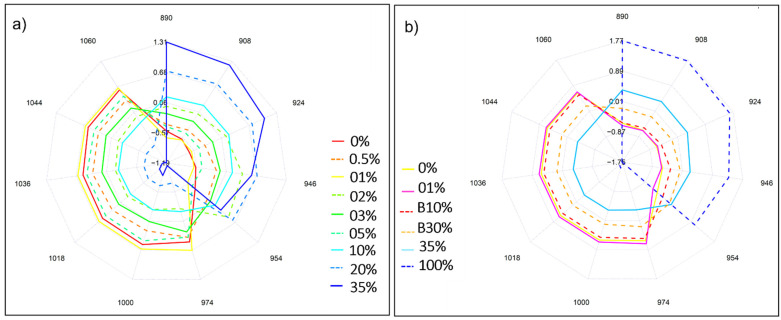
(**a**) Aquagrams of aqueous solutions of Robusta-Arabica blends in the concentration range of 0.5–35% and pure Arabica (0%) in the second overtone region; (**b**) Aquagram of the dataset comprising pure Arabica (0%), pure Robusta (100%), Robusta-Arabica blends (01% and 35%) and marketed blends (B10% and B30%).

The complexity of differentiating between the mixtures with the lowest adulteration levels was evidenced primarily by their respective water spectral pattern where an overlapping of blends containing Robusta fractions as low as 0.5%, 1%, and 2% occurred. Notably, above these concentration levels, the higher the ratio Robusta to Arabica was, the higher the absorbance in the wavelengths that are characteristic of pure Robusta. Inversely, the lower the added Robusta, the higher the absorbance in the wavelengths characteristic of pure Arabica (Figure 9a).

Assessing whether or not the inclusion of marketed blends B10% and B30% can still be translated into distinctive water spectral patterns, in the presence of lab generated mixtures covering both low (1%) and high blending ratios (35%), was also attempted and confirmed the adequacy of the analysis from an aquaphotomics standpoint in terms of highlighting the respective composition of the studied samples. Once again, the intricacy of detecting the lowest blending ratio was reflected by a slight overlapping with pure Arabica extract (Figure 9b).

## 3. Materials and Methods

### 3.1. Samples Preparation

For the purpose of our study, Arabica beans originating from Brazil (ARA1), Columbia (ARA2), and Ethiopia (ARA3) and Robusta beans sourced from Vietnam (ROB1), Uganda (ROB2), and India (ROB3) were French-roasted, ground, and procured by Bourbon café (Tahitótfalu, Hungary). Mixtures comprising 0.5, 1, 2, 3, 5, 10, 20, 35% *w*/*w* Robusta were prepared by pairing ARA3 and ROB3. The threshold above which the addition of Robusta can have a palpable effect on coffee aroma is 35% [31]. Alongside these mixtures, marketed blends of different provenance and serving as test samples were considered. The 1st blend B10% consisted of 90% Arabica (South American) and 10% Robusta (South-east Asian), whereas the 2nd blend B30% comprised 70% Arabica (Central and South American) and 30% Robusta (Southeast Asian). Triplicate samples were prepared for each of the mixture concentration levels.

After formulating the mixtures, water extracts of the pure coffee varieties, the mixtures, as well as the marketed blends were prepared by pouring 100 mL Milli-Q water, heated at boiling point, onto 8 g of coffee. After five minutes, the samples were filtered using a 25 µm pore-sized quantitative filter Whatman paper. The obtained extracts were cooled to room temperature (25 °C) prior to analysis.

### 3.2. Instrumental Analysis

A benchtop MetriNIR Spectrometer (MetriNIR Research, Development and Service Co., Budapest, Hungary) was used to collect the spectral data in the wavelength range of 740–1700 nm. For a more representative spectra of each of the ground coffee mixtures, the cuvette was rotated between the three consecutive scans of each sample type during scanning.

In the case of the aqueous samples, a thermoregulated cuvette with a sample layer thickness of 0.5 mm was used to maintain the temperature of the samples at 25 °C. The cuvette was thoroughly washed with Milli-Q water between measurements and dried for the next sample. A total of 324 spectra, made up of three consecutive scans of the three refills of the triplicates of each mixture, were acquired. For reference data, Milli-Q water spectra were taken after every 5th sample measurement.

Two modes of spectra acquisition were adopted, diffuse reflectance mode in the case of powders and transflectance mode for liquids.

### 3.3. Data Processing

The selected spectral ranges were the truncated 800–1670 nm region, used to reduce spectral noise, as well as the first (1300–1600 nm) and second (800–1100 nm) overtone regions of water (more specifically OH bond), necessary for the aquaphotomics based analysis [17,32].

A set of spectral preprocessing methods were tested in terms of their effect on the obtained results. Initially, an essential step was to conduct spectral smoothing using Savitzky Golay filter by fitting the spectral points into a 2nd polynomial within the selected window width (11, 17, or 19 points). The smoothing was also jointly applied with one of the following pretreatments: multiplicative scatter correction (MSC), detrend (DeTr), standard normal variate (SNV), 1st or 2nd derivative.

The chemometric tools used for the statistical analysis of the multivariate data consisted mostly of principal component analysis (PCA), used as a pattern recognition and dimensionality reduction tool. In addition, hybrid principal component analysis-linear discriminant analysis (PCA-LDA) models served as multi-class classifiers. Ensuring that the number of PCs was optimal for further modeling was essential. In doing so, the PCs guaranteeing a combination of the best validation accuracy and the minimal difference between training and validation accuracies were selected. The dataset was split into a calibration set (two thirds) and a validation set (one third) and the three-fold cross validation (CV) method was used to assess the predictive accuracy of the PCA-LDA model in classifying pure Arabica and Robusta, their resulting mixtures at different blending ratios, as well as the marketed blends.

Subsequently, to relate the NIR spectrum to the added Robusta, partial least squares regression modelling (PLSR) was performed. Different validation methods were considered before deciding upon the model that ensures the most accurate prediction of the Robusta-to-Arabica ratio. The evaluation of the PLS models was done by computing the coefficient of determination (R^2^) and the root mean square error (RMSE) of both the calibration and cross-validation. These metrics assess the fit of the tested data to the regression line while estimating the difference between predicted and actual values. The closer R^2^ is to 1 and the lower RMSEC is, the more accurate the model [33]. As per the validation methods, they ranged from the least robust leave one sample out validation to more robust cross-validation based on the grouping defined by specific class variables (by repeats, by sample type, etc.). Testing the accuracy of the predictive models was performed using the two commercialized blends B10% and B30%. Only the best models were displayed in the present manuscript.

Aquagrams, representative tools of the water spectral pattern, were investigated both at the 1st and 2nd overtone regions at selected water matrix coordinates (WAMACs). These WAMACs consisted of the following wavelengths: 890, 908, 924, 946, 954, 975, 1001, 1019, 1036, 1044, and 1060 nm in case of the 2nd overtone and involved 1342, 1364, 1374, 1384, 1412, 1426, 1440, 1452, 1462, 1476, 1488, and 1512 nm when plotted in the 1st overtone range [34]. Based on literature, these wavelengths have been assigned to specific water molecular structures and have demonstrated their practicality with regard to highlighting the effect of certain perturbations on the studied system [17,19,22]. While both of the overtone ranges (1st and 2nd) were considered, the one portraying the most water spectral pattern differences between the studied samples corresponded to the 2nd overtone region and was the one presented in the manuscript.

In order to minimize sources of variations within each sample type, averaging of the repeats, refills, and consecutives was also attempted throughout the analysis.

## 4. Conclusions

To date, coffee adulteration issues have been addressed extensively, however; few are the studies that were not based on complex coffee extraction methods. Even fewer studies did not resort to chemical markers for the differentiation of the two coffee varieties (Arabica and Robusta) in liquid state.

Our study sought to extend the research done on coffee quality analysis through the application of both conventional NIR spectroscopy and its novel approach, aquaphotomics.

Conventional spectroscopy provided satisfactory results in terms of distinguishing pure Arabica and Robusta ground coffee from different geographical origins with 100% correct classification accuracy. When mixtures of these varieties were prepared by varying the blending ratios, accurate classification and quantification models were achieved depending on the Robusta to Arabica content. Only 5.55% of the ground coffee mixtures were misidentified by means of LDA analysis. As per the prediction of the Robusta-to-Arabica ratio, it was estimated with R^2^CV of 0.99 and an error (RMSECV) of 2.4% *w*/*w*.

On the other hand, implementing aquaphotomics-based research was found to give typical spectral fingerprints to the aqueous blends. As the added Robusta increased, the corresponding mixtures had higher absorbances in the wavelengths associated with pure Robusta. A prominence of bonds attributed to water shells, as well as V1 and V2 bonded water was featured in their respective aquagrams. Conversely, those with higher Arabica fraction presented characteristic spectral patterns at the WAMACS linked to pure Arabica. The marked spectral features, in the latter case, were mainly attributed to an abundance of high hydrogen bonded water structures and water clusters with two, three, and four hydrogen bonds. The efficacy of the implemented approach was further corroborated when marketed blends were examined, correctly discriminated, and their Robusta contents accurately estimated (R^2^CV of 97% and 90% by NIRS and aquaphotomics respectively). 

What the findings of this study pinpoint is that aquaphotomics could be a suitable, cost-effective alternative to other technically demanding authentication tools. Most importantly, it offers novel insights regarding the changes of the water spectral pattern induced by the increased amount of Robusta. If implemented, this approach could reveal undeclared blending of coffee and contribute to the detection of potential adulteration. Nevertheless, it is worth pointing out that the applied method requires further refining with the inclusion of other coffee mixtures prepared under different roasting and brewing processes.

## Figures and Tables

**Table 1:** PCA-LDA classification model on the NIRS data of pure and adulterated mixtures after three-fold cross-validation (N = 268).

Validation Accuracy %											
Robusta-to-Arabica Ratio											
		0%	0.5%	1%	2%	3%	5%	10%	20%	35%	100%
**Robusta-to-Arabica ratio**	**0%**	**66.74**	8.03	0	7.44	0	11.11	11.11	0	0	0
	**0.5%**	14.79	**51.92**	7.45	7.44	14.78	7.44	7.44	0	3.67	0
	**1%**	0	3.96	**70.41**	0	7.44	0	0	11.11	0	0
	**2%**	0	8.03	0	**81.44**	7.44	0	0	0	0	0
	**3%**	3.67	20.02	3.67	0	**55.56**	7.44	3.67	0	0	0
	**5%**	3.67	0	3.67	3.67	11.11	**66.67**	3.67	7.44	3.67	0
	**10 %**	7.45	8.03	0	0	3.67	3.67	**66.67**	7.44	3.67	0
	**20%**	3.67	0	14.79	0	0	3.67	7.44	**70.33**	3.67	0
	**35%**	0	0	0	0	0	0	0	3.67	**85.32**	0
	**100%**	0	0	0	0	0	0	0	0	0	**100**

## Data Availability

The data presented in this study are available on request from the corresponding author. The data are not publicly available, due to privacy reasons.
